# Adverse dengue outcomes in patients with chronic kidney disease: A population-based analysis of 5.8 million individuals from Brazil

**DOI:** 10.1371/journal.pntd.0013927

**Published:** 2026-01-20

**Authors:** Ricardo Augusto Monteiro de Barros Almeida, Leticia Lastória Kurozawa, Gabriel Berg de Almeida, Ricardo de Souza Cavalcante, Raoni de Oliveira Domingues-da-Silva, Elizabeth De Francesco Daher, Douglas Otomo Duarte, Luis Gustavo Modelli de Andrade

**Affiliations:** 1 Department of Infectious Diseases, Dermatology, Imaging Diagnosis, and Radiotherapy. Sao Paulo State University (UNESP). Botucatu, Sao Paulo, Brazil,; 2 Hospital Epidemiology Center, Sao Paulo State University (UNESP). Botucatu, Sao Paulo, Brazil,; 3 Internal Medicine Department, Federal University of Ceará, Fortaleza, Ceará, Brazil; 4 Department of Internal Medicine, Sao Paulo State University (UNESP). Botucatu, Sao Paulo, Brazil; The University of the West Indies, JAMAICA

## Abstract

**Background:**

Dengue fever poses a major public health threat in Brazil, particularly among individuals with comorbid conditions. Chronic kidney disease (CKD) is known to impair immune defenses and may predispose patients to worse infectious outcomes, but its impact on dengue has not been comprehensively evaluated at the national level. This study aimed to assess the association between CKD and adverse clinical outcomes of dengue fever.

**Methods:**

We conducted a population-based analysis using 2024 data from the Brazilian Information System for Notifiable Diseases (SINAN). Patients with a confirmed diagnosis of dengue, based on laboratory or clinical-epidemiological criteria, were included in the study. The cohort was stratified based on their CKD status. Clinical features and outcomes (hospitalization, severe dengue, and death) were compared. Multivariable logistic regression models weighted by inverse probability of treatment weighting (IPTW) were used to estimate adjusted odds ratios (ORs).

**Results:**

Among 5,837,904 confirmed dengue cases, 30,527 (0.5%) had CKD. Compared to patients without CKD, those with CKD had significantly higher rates of hospitalization (14% vs. 4%), severe dengue (1.6% vs. 0.1%), and death (2.2% vs. 0.1%) (all p < 0.001). In adjusted analyses, CKD was independently associated with increased odds of death (OR = 3.09, 95% CI: 2.66–3.59) and the composite outcome (OR = 1.62, 95% CI: 1.53–1.71). Hematologic and autoimmune comorbidities also conferred increased risk.

**Conclusion:**

CKD significantly worsens clinical outcomes in dengue fever. These findings highlight the need for early recognition, tailored care, and targeted public health strategies to protect this high-risk population during dengue outbreaks.

## Author summary

Dengue affects millions of people in Brazil every year, but not everyone faces the same risk of severe illness. We asked a simple question with big public-health consequences: do people living with chronic kidney disease have worse outcomes when they get dengue? To answer it, we examined Brazil’s national notifiable-disease records for all confirmed dengue cases in 2024. We compared those with and without chronic kidney disease and accounted for differences such as age and other health problems. We found a clear pattern. People with chronic kidney disease were hospitalized more often, progressed to severe dengue more often, and had a markedly higher risk of death, about three times higher, than those without kidney disease. Warning signs that indicate possible complications were also more frequent in this group. These findings matter for everyday care and for planning during outbreaks. They support prioritizing people with chronic kidney disease for early evaluation, careful fluid management, and timely referral. They also strengthen the case for including this group among those prioritized for dengue vaccination in endemic areas. Our study provides national-level evidence to help clinicians and health authorities protect a highly vulnerable population.

## Introduction

Dengue fever is the most common arboviral disease globally, with its burden disproportionately concentrated in tropical and subtropical regions. In Latin America, Brazil consistently accounts for the highest number of reported cases, reflecting both endemic transmission and recurrent epidemic waves driven by climatic and ecological conditions [1]. Brazil’s 2024 dengue epidemiology features the co-circulation of all four serotypes (DENV-1–4), with a pronounced prevalence of DENV-1 and DENV-2 [2]. The Brazilian Information System for Notifiable Diseases (SINAN) serves as a vital surveillance tool, enabling the systematic collection of clinical and epidemiological data on dengue cases nationwide [3].

Most dengue infections are self-limiting; however, a subset can progress to severe forms of the disease. These severe cases are characterized by hemorrhagic manifestations, plasma leakage, circulatory failure, and, in some instances, organ dysfunction [4]. To aid in the clinical management of dengue, the World Health Organization (WHO) classifies non-severe symptomatic dengue cases into two categories: dengue with warning signs and dengue without warning signs. Warning signs include abdominal pain or tenderness, persistent vomiting, clinical fluid accumulation, mucosal bleeding, lethargy, restlessness, liver enlargement greater than 2 cm, and an increase in hematocrit (HCT) concurrent with a rapid decrease in platelet count [4]. Patients exhibiting warning signs need close monitoring, as they are more likely to progress to severe disease. Severe dengue is defined by the presence of any of the following clinical manifestations: severe plasma leakage resulting in shock or fluid accumulation with respiratory distress, severe bleeding, or significant organ impairment such as hepatitis (indicated by elevated transaminases ≥1,000 IU/L), impaired consciousness, or heart impairment [4].

Host comorbidities have been increasingly recognized as important modulators of dengue severity. Conditions such as diabetes mellitus, hypertension, and chronic kidney disease (CKD) are particularly linked to impaired immune responses and vascular dysfunction, which may increase the risk of severe outcomes in dengue patients [5,6].

However, the specific impact of CKD on dengue-related outcomes remains poorly understood, especially in large national cohorts. Given the growing prevalence of CKD and its association with increased susceptibility to infections and immune dysregulation [7,8], it is critical to investigate its role in the clinical trajectory of dengue.

To address this gap, we conducted a nationwide cohort study using the 2024 SINAN database to investigate dengue-related outcomes among patients with and without CKD. We hypothesized that individuals with CKD would experience significantly worse clinical outcomes, including higher rates of hospitalization, severe dengue, and death. To minimize potential confounding, we applied inverse probability of treatment weighting (IPTW) based on sociodemographic and clinical variables.

## Methods

### Study design and data source

We used data from the Information System for Notifiable Diseases (SINAN), the official Brazilian national surveillance system for reportable diseases. SINAN collects standardized demographic, clinical, and laboratory information on suspected and confirmed dengue cases across Brazil. For this study, we included only cases with a confirmed diagnosis of dengue fever reported during calendar year 2024, resulting in a large cohort. Confirmation was based on either laboratory criteria or clinical-epidemiological linkage, as per national reporting guidelines [9]. A detailed mapping of the clinical and laboratory parameters used to define confirmed dengue in the database, and their correspondence with guideline-based criteria, is provided in S1 Table.

### Study population

We included all individuals with confirmed dengue fever who had complete information on the presence or absence of CKD. CKD status was identified using a specific field within the notification form. We excluded notifications classified as discarded or still under investigation, those without a defined confirmation criterion, and those with missing data on CKD or other key covariates.

### Exposure, covariates, and outcomes

The primary exposure variable was chronic kidney disease (CKD), as documented in the comorbidity section of the SINAN dengue notification form, which includes a specific checkbox completed by the reporting health professional. In Brazilian clinical practice, CKD is generally defined in accordance with international guidelines as abnormalities of kidney structure or function for ≥3 months, typically an eGFR < 60 mL/min/1.73 m^2^ and/or markers of kidney damage such as albuminuria; however, these laboratory data and the duration of kidney dysfunction are not captured in SINAN. Therefore, in this study CKD represents a clinician-reported pre-existing diagnosis rather than a uniform eGFR-based definition.

Covariates considered in the analysis included sociodemographic characteristics such as age, sex, race or ethnicity (categorized as White, Black or Multiracial Black and White, and Yellow or Indigenous), geographic macro-region of residence (North, Northeast, Southeast, South, or Central-West), and educational attainment. The method of diagnostic confirmation was also included as a covariate and was classified as either laboratory-confirmed or based on clinical-epidemiological criteria.

Clinical presentation variables included the presence or absence of key dengue-related symptoms such as fever, myalgia, headache, rash, vomiting, nausea, back pain, conjunctivitis, arthritis, arthralgia, petechiae, retro-orbital pain, and a positive tourniquet test, as well as the presence of leukopenia. In addition, we accounted for coexisting medical conditions, diabetes mellitus, hematologic disorders, hypertension, and autoimmune diseases, as reported in the notification forms.

Dengue with warning signs was defined as dengue presenting with one or more clinical warning signs, including abdominal pain or tenderness, persistent vomiting, clinical fluid accumulation, mucosal bleeding, lethargy, restlessness, faintness, orthostatic hypotension, liver enlargement > 2 cm, an increase in hematocrit (HCT), and concurrent with a rapid decrease in platelet count. Severe dengue was defined by dengue with any of the following clinical manifestations: severe plasma leakage leading to shock or fluid accumulation with respiratory distress; severe bleeding; or severe organ impairment such as hepatitis (elevated transaminases ≥1,000 IU/L), impaired consciousness, or heart impairment [4,9].

The primary outcomes of interest were hospitalization, severe dengue, and death. To comprehensively assess the clinical impact of dengue infection, we also defined a composite outcome of severe disease, characterized by the occurrence of at least one of the following events: hospitalization, severe dengue, or death.

### Ethical considerations

This study did not require approval from a Research Ethics Committee, as it is based on publicly available data from an official national database (SINAN) that contains aggregated, de-identified information. The data was available at: https://portalsinan.saude.gov.br/.

### Statistical analysis

Clinical characteristics and outcomes were compared between individuals with and without CKD. Categorical variables were compared using chi-squared tests, while continuous variables, reported as medians with interquartile ranges (IQR), were compared using the Wilcoxon rank-sum test due to non-normal distributions.

To reduce confounding arising from baseline differences between patients with and without CKD, we applied inverse probability of treatment weighting (IPTW) based on a propensity score [10]. Propensity scores were estimated using logistic regression, with CKD status as the dependent variable and the following covariates included: age, sex, race/ethnicity, geographic region, education level, and diagnostic method (laboratory vs. clinical-epidemiological). To mitigate the influence of extreme weights, values were truncated at the 99^th^ percentile. Covariate balance before and after weighting was assessed using standardized mean differences (SMDs), with an SMD < 0.1 considered indicative of acceptable balance. Balance diagnostics were performed using the cobalt package in R.

Stabilized weights were calculated as the ratio of the marginal probability of CKD to the estimated propensity score for individuals with CKD, and the ratio of the marginal probability of not having CKD to one minus the propensity score for individuals without CKD. These weights were then applied in weighted logistic regression models to estimate the association between CKD and the primary outcome (mortality), as well as a composite outcome comprising hospitalization, severe dengue, or death.

The use of IPTW provides doubly robust estimates, meaning valid inference can be achieved if either the propensity score model or the outcome model is correctly specified. This approach is increasingly recognized in real-world comparative effectiveness research for its ability to reduce bias due to observed confounding [10].

All analyses were performed using R software (version 4.3). A two-sided p-value of less than 0.05 was considered statistically significant.

### Role of the funding source

There was no funding source for this study.

## Results

**Fig 1:**
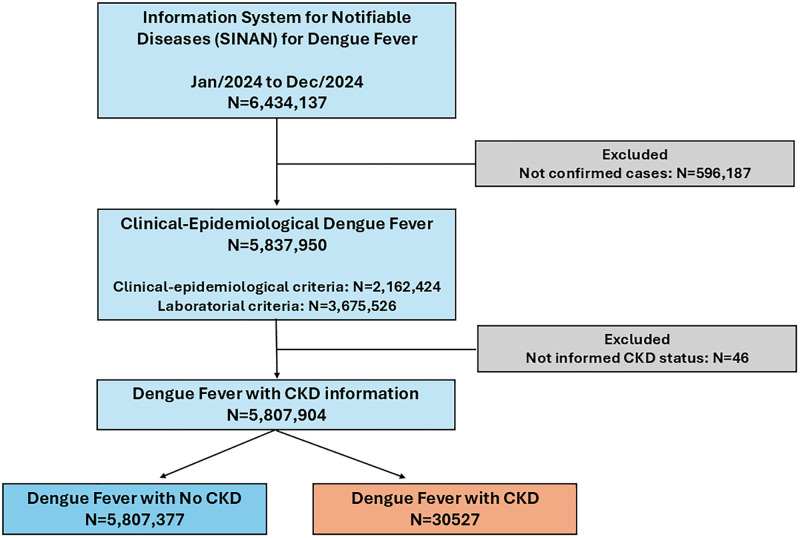
Flowchart of case selection from the Brazilian Information System for Notifiable Diseases (SINAN) for dengue fever, 2024.

We used data from the Brazilian Information System for Notifiable Diseases (SINAN) on dengue fever cases reported between January and December 2024. A total of 6,434,137 records were retrieved. After excluding 596,187 cases not confirmed by laboratory or clinical-epidemiological criteria, 5,837,950 confirmed dengue cases remained eligible. Of these, 46 cases were excluded due to missing information on chronic kidney disease (Fig 1). Out of 5,837,904 confirmed dengue cases, 30,527 patients (0.5%) had CKD. Compared to patients without CKD, those with CKD were markedly older (median age 49 [IQR: 30–66] vs. 35 [IQR: 21–52] years) and more frequently reported comorbidities. The prevalence of diabetes was substantially higher among CKD patients (40% vs. 4.3%), as was hypertension (52% vs. 10%). Hematologic disorders (26% vs. 0.4%) and autoimmune diseases (24% vs. 0.6%) were also more common in the CKD group (Table 1).

**Table 1:** Baseline characteristics of patients with confirmed dengue, stratified by presence of chronic kidney disease (CKD).

	Overall (n = 5,837,904)	Non-CKD (n = 5,807,377)	CKD (n = 30,527)	*p-value*
Demographics				
Sex				0.9
Female	3,185,442 (55%)	3,168,769 (55%)	16,673 (55%)	
Male	2,645,325 (45%)	2,631,505 (45%)	13,820 (45%)	
Unknown	7,137	7,103	34	
Age				<0.001
Age	35 (21–52)	35 (21–52)	49 (30–66)	
Unknown	23,632	23,462	140	
Race/ethnicity				<0.001
Black/Multiracial	2,280,231 (46%)	2,268,345 (46%)	11,886 (44%)	
Black and White				
White	2,566,103 (52%)	2,551,692 (52%)	14,411 (54%)	
Yellow/Indigenous	66,717 (1.4%)	66,280 (1.4%)	437 (1.6%)	
Unknown	924,853	921,060	3,793	
Education				<0.001
Basic Education	773,196 (32%)	767,680 (32%)	5,516 (39%)	
Higher Education	320,098 (13%)	318,342 (13%)	1,756 (12%)	
Illiterate	21,404 (0.9%)	21,109 (0.9%)	295 (2.1%)	
Secondary Education	1,289,593 (54%)	1,282,833 (54%)	6,760 (47%)	
Unknown	3,433,613	3,417,413	16,200	
Region				<0.001
Central-West	572,703 (10%)	569,920 (10%)	2,783 (9.4%)	
North	49,800 (0.9%)	49,581 (0.9%)	219 (0.7%)	
Northeast	80,632 (1.4%)	80,348 (1.4%)	284 (1.0%)	
South	1,155,235 (20%)	1,148,652 (20%)	6,583 (22%)	
Southeast	3,812,140 (67%)	3,792,468 (67%)	19,672 (67%)	
Unknown	167,394	166,408	986	
Symptoms/signs				
Fever	5,008,100 (86%)	4,982,311 (86%)	25,789 (84%)	<0.001
Muscle Pain	4,665,411 (80%)	4,641,259 (80%)	24,152 (79%)	<0.001
Headache	4,690,166 (80%)	4,666,373 (80%)	23,793 (78%)	<0.001
Rash	574,658 (9.8%)	569,778 (9.8%)	4,880 (16%)	<0.001
Vomiting	1,601,648 (27%)	1,590,895 (27%)	10,753 (35%)	<0.001
Nausea	2,607,311 (45%)	2,590,350 (45%)	16,961 (56%)	<0.001
Back Pain	1,814,239 (31%)	1,800,539 (31%)	13,700 (45%)	<0.001
Conjunctivitis	232,370 (4.0%)	228,276 (3.9%)	4,094 (13%)	<0.001
Arthritis	554,993 (9.5%)	548,537 (9.4%)	6,456 (21%)	<0.001
Joint Pain	994,934 (17%)	986,747 (17%)	8,187 (27%)	<0.001
Petechiae	326,830 (5.6%)	322,159 (5.5%)	4,671 (15%)	<0.001
Leukopenia	229,403 (3.9%)	225,067 (3.9%)	4,336 (14%)	<0.001
Tourniquet Test	201,431 (3.5%)	197,710 (3.4%)	3,721 (12%)	<0.001
Retroorbital Pain	1,902,470 (33%)	1,890,592 (33%)	11,878 (39%)	<0.001
Comorbidities				
Diabetes	262,336 (4.5%)	250,032 (4.3%)	12,304 (40%)	<0.001
Hematologic disorders	32,489 (0.6%)	24,498 (0.4%)	7,991 (26%)	<0.001
Hypertension	604,732 (10%)	588,725 (10%)	16,007 (52%)	<0.001
Autoimmune disease	41,924 (0.7%)	34,499 (0.6%)	7,425 (24%)	<0.001
Diagnostic				
Diagnostic Criteria				<0.001
Clinical-Epidemiological	3,675,490 (63%)	3,657,539 (63%)	17,951 (59%)	
Laboratory	2,162,414 (37%)	2,149,838 (37%)	12,576 (41%)	

Data are median (IQR) or n (%).

The symptom profile of dengue differed between groups (Table 1). Among the differences in symptoms, we can highlight higher frequencies in CKD group of cutaneous rash (16% vs. 9.8%), nausea (56% vs. 45%), vomiting (35% vs. 27%), back pain (45% vs. 31%), arthritis (21% vs. 9.4%), joint pain (27% vs. 17%), retro-orbital pain (39% vs. 33%), petechiae (15% vs. 5.5%), and conjunctivitis (13% vs. 3.9%). Leukopenia (14% vs. 3.9%) and a positive tourniquet test (12% vs. 3.4%) were also more frequent in CKD patients.

Outcomes were significantly worse among individuals with CKD (Table 2). Hospitalization occurred in 14% of CKD patients compared to 4% of those without CKD (p < 0.001). Severe dengue was reported in 1.6% of CKD cases versus only 0.1% of non-CKD cases (p < 0.001). The crude mortality rate was 2.2% in the CKD group, compared to 0.1% in patients without CKD (p < 0.001). Warning signs of dengue were reported in 5.7% of CKD cases, compared to only 1.8% of non-CKD cases (p < 0.001).

**Table 2:** Clinical outcomes of patients with dengue fever, stratified by presence of chronic kidney disease (CKD), Brazil 2024.

	Overall (n = 5,837,904)	Non-CKD (n = 5,807,377)	CKD (n = 30,527)	*p-value*
Death	7,665 (0.1%)	7,084 (0.1%)	581 (2.2%)	<0.001
Unknown	636,277	632,734	3,543	
Hospitalization	175,509 (4.0%)	172,207 (4.0%)	3,302 (14%)	<0.001
Unknown	1,501,647	1,493,946	7,701	
Severe dengue	9,501 (0.2%)	8,576 (0.1%)	475 (1.6%)	<0.001
Composite^*^	178,312 (4.5%)	174,938 (4.4%)	3,374 (16%)	<0.001
Unknown	1,841,184	1,831,675	9,509	
Warning signs	108,352 (1.9%)	106,625 (1.8%)	1,727 (5.7%)	<0.001

* Composite outcome of death, hospitalization, or severe dengue.

**Fig 2:**
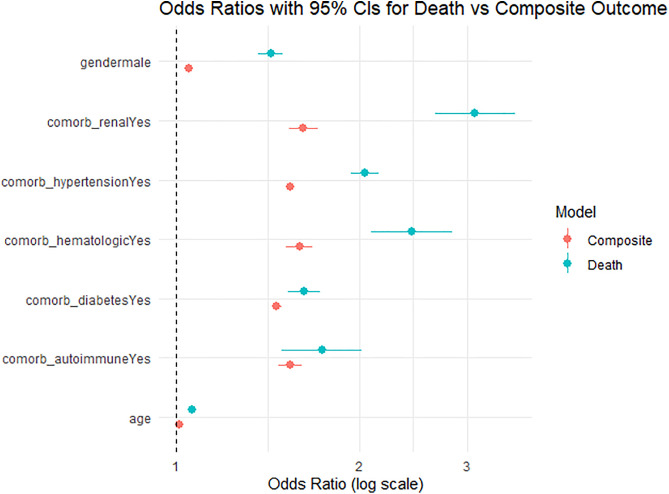
Adjusted Odds Ratios for Adverse Clinical Outcomes in Dengue Fever According to Patient Comorbidities. This figure displays adjusted odds ratios (ORs) and 95% confidence intervals for key clinical and demographic variables associated with two outcomes in patients with confirmed dengue fever in Brazil during 2024: death (red) and a composite outcome including death, hospitalization, or progression to severe dengue (blue). Estimates were derived from multivariable logistic regression models weighted using inverse probability of treatment weighting with stabilization (IPWS) to control for sociodemographic variables and diagnostic method. Chronic kidney disease, hematologic disorders, and autoimmune conditions were consistently associated with increased odds of both outcomes. A dashed vertical line at OR = 1.0 indicates the null effect.

After applying inverse probability of treatment weighting (IPTW) to adjust for sociodemographic variables and diagnostic method, multivariable logistic regression demonstrated that CKD was independently associated with a significantly increased risk of both death (OR = 3.09, 95% CI: 2.66–3.59, p < 0.001) and the composite outcome of death, hospitalization, or severe dengue (OR = 1.62, 95% CI: 1.53–1.71, p < 0.001) (Table 3). Other comorbidities also showed strong associations with adverse outcomes. Age, male sex, diabetes, hematologic diseases, hypertension, and autoimmune conditions were also significant predictors of both mortality and the composite outcome (Table 3 and Fig 2).

**Table 3:** Multivariable logistic regression for factors associated with death, and composite outcome (death, hospitalization, or severe dengue) among dengue patients in Brazil, 2024.

	Death OR 95%CI, p-value	Composite Outcome OR 95%CI, p-value
Age	1.06 (1.06–1.06), p < 0.001	1.01 (1.01–1.01), p < 0.001
Male	1.43 (1.37–1.50), p < 0.001	1.05 (1.04–1.06), p < 0.001
Diabetes	1.62 (1.53–1.72), p < 0.001	1.46 (1.44–1.49), p < 0.001
Hematologic disorder	2.43 (2.09–2.84), p < 0.001	1.59 (1.52–1.67), p < 0.001
Chronic kidney disease	3.09 (2.66–3.59), p < 0.001	1.62 (1.53–1.71), p < 0.001
Hypertension	2.04 (1.93–2.15), p < 0.001	1.54 (1.51–1.56), p < 0.001
Autoimmune disease	1.74 (1.49–2.02), p < 0.001	1.54 (1.47–1.61), p < 0.001

Multivariable logistic regression models were adjusted using inverse probability of treatment weighting (IPTW) to account for differences in sociodemographic characteristics (age, gender, race, region, and education) and diagnostic method.

Additionally, a multivariable logistic regression demonstrated that CKD was independently associated with a significantly increased risk of severe dengue (OR = 2.50, 95% CI: 2.12–2.94, p < 0.001), dengue with warning signs (OR = 1.26, 95% CI: 1.17–1.35, p < 0.001), and hospitalization (OR = 1.61, 95% CI: 1.52–1.70, p < 0.001).

## Discussion

This study provides compelling evidence that CKD is independently associated with significantly worse clinical outcomes in patients with dengue fever, including markedly higher risks of hospitalization, progression to severe dengue, and mortality. These findings are consistent with prior studies indicating that individuals with CKD are more susceptible to severe outcomes from infectious diseases due to impaired innate and adaptive immune responses, chronic systemic inflammation, and a higher burden of comorbidities such as diabetes and hypertension [5–8].

A large multicenter observational study identified CKD, along with advanced age and leukocytosis, as an independent predictor of in-hospital mortality among adults hospitalized with dengue. In patients with CKD who developed dengue, mortality was high, with nearly half of CKD patients succumbing to the infection, and a substantial proportion dying within the first week of illness. Patients with CKD and dengue virus infection had 3**.**9 times higher odds of death, closely aligning with our findings. Among CKD patients with dengue, initial altered consciousness, pulmonary edema, and leukocytosis during hospitalization were independently associated with increased risk of death, and severe hepatitis further increases the risk of early mortality [11].

A study conducted in Ceará, northeastern Brazil, from January 2015 to December 2017 included a total of 161,880 patients and utilized the same database as the present study. The findings revealed that mortality rates were significantly higher in patients with CKD, with the rate of 2.2% compared to 0.1% for those without CKD (p < 0.001). Furthermore, CKD was recognized as an independent risk factor for mortality, with an odds ratio of 3.96 (95% confidence interval: 2.09–7.54) [12]. The data from this study closely resembled the findings from the present research.

A systematic review and meta-analysis investigated 262 global dengue outbreaks from 1990 to 2015 [5]. The results showed that patients with renal impairment had an odds ratio of 5**.**26 for developing dengue hemorrhagic fever. Additionally, another systematic review and meta-analysis by Tsheten et al., which included 143 studies, estimated a pooled odds ratio of 4.54 (95% CI: 1.55–13.31) for the development of severe dengue in patients with renal disease, including CKD [13]. Our study showed a lower odds ratio, which may be attributed to varying definitions of renal disease stages. Nonetheless, the heightened risk of severe dengue in patients with CKD, as indicated by the systematic reviews and meta-analyses, supports our findings.

Chen et al. [14] demonstrated that 36.7% of patients with end-stage renal disease undergoing dialysis who developed dengue infection exhibited clinical warning signs. Notably, individuals presenting warning signs had a sevenfold increased risk of progressing to severe dengue compared to those without such signs. Consistent with these findings, the elevated frequency of warning signs among individuals with CKD observed in our cohort underscores the heightened vulnerability of this population to severe clinical manifestations of dengue.

Two national cohort studies have investigated CKD as a risk factor for adverse clinical outcomes in patients with dengue. The first, conducted in Taiwan, followed 51,433 laboratory-confirmed adult dengue cases over a 30-day period between 2014 and 2015 [15]. The findings indicated that individuals with CKD had significantly higher odds of hospitalization (OR: 1.49), intensive care unit (ICU) admission (OR: 2.41), and all-cause mortality (OR: 3**.**03), underscoring the increased clinical vulnerability of this population. Additionally, patients with CKD experienced a 19.3% increase in average length of hospital stay and a 94.7% increase in mean total medical expenditures. A second investigation—a nationwide retrospective cohort study conducted in Mexico—analyzed data from 18,436 laboratory-confirmed dengue cases reported between 2020 and mid-2024 [16]. The study reported a mortality rate of 4.9% among individuals with CKD and identified CKD as a significant independent risk factor for mortality, with a relative risk (RR) of 3.35. Collectively, these findings reinforce the associations observed in the present study and underscore the heightened vulnerability of CKD patients during dengue outbreaks.

Dengue infection in CKD and ESRD patients is characterized by impaired immune responses. In vitro studies demonstrate that mononuclear cells from ESRD patients infected with dengue virus exhibit dysregulated cytokine production, with lower levels of key immune mediators such as IL-8, IL-10, IL-12p40, TNF-α, MCP-1, and MIP-1b compared to healthy controls. This immunological impairment may contribute to the increased susceptibility to severe dengue and poorer outcomes in this population [17].

The pathophysiology of renal involvement in dengue fever encompasses several factors, including the direct effects of the virus, immune-mediated injury, rhabdomyolysis, and hemodynamic instability. These issues can be aggravated in individuals with pre-existing CKD [18,19]. Endothelial cell dysfunction in the uremic environment may explain the more severe hemoconcentration observed in CKD patients [20]. Additionally, the cytokine storm in CKD patients may contribute to the capillary leak syndrome associated with dengue fever [21]. Furthermore, the hesitation to administer extra fluids for hydration during the febrile illness in CKD patients—due to the concern about potential fluid overload—may also lead to increased hemoconcentration in this group [18].

Clinically, dengue infection in CKD patients is associated with a higher incidence and severity of acute kidney injury, more frequent and severe worsening of renal function, and a greater need for renal replacement therapy compared to patients with normal baseline renal function. In one prospective cohort, all CKD patients and most kidney transplant recipients with dengue experienced worsening renal function, and a significant proportion of CKD patients required dialysis, with some remaining dialysis-dependent at two weeks post-infection. In contrast, worsening renal function was less severe and more transient in renal transplant recipients [18].

The strength of this analysis lies in the unprecedented scale of the dataset, comprising over 5**.**8 million confirmed dengue cases reported across Brazil in 2024. This extensive sample enabled a highly powered and nationally representative comparison between patients with and without CKD. Furthermore, the application of inverse probability of treatment weighting (IPTW) allowed for adjustment of key sociodemographic and diagnostic variables, thereby emulating a pseudo-randomized design and enhancing the robustness of causal inferences derived from observational data [22].

In our cohort, the frequency of CKD recorded in SINAN was lower than would be expected based on population-based estimates of CKD in Brazil, which range from about 1–2% using self-reported diagnosis in the National Health Survey to approximately 6–7% when laboratory criteria are applied, and can exceed 10% in regional studies using eGFR and albuminuria [23–25]. This discrepancy most likely reflects characteristics of the surveillance data rather than a truly low burden of kidney disease among patients with dengue. First, dengue notifications in SINAN are concentrated in younger adults and children, age strata with intrinsically lower CKD prevalence than the older populations from which most national CKD estimates are derived. Second, CKD is captured only as a single clinician-reported comorbidity field without laboratory parameters or information on the duration of kidney dysfunction. Consequently, CKD is likely under-ascertained, particularly among individuals with undiagnosed CKD or early stages of kidney damage, and our estimates should be interpreted as a subset of clinically recognized CKD rather than the true prevalence of CKD among people with dengue.

The study has some limitations that merit consideration. First, the SINAN database depends on routine clinical reporting, and CKD status is either self-reported or based on clinical documentation without standardized diagnostic validation. This may result in misclassification bias or underreporting, particularly among individuals with early-stage or undiagnosed CKD. Additionally, critical clinical information, such as CKD stage, dialysis dependence, etiology, and duration of kidney disease, is not captured, limiting stratified risk analyses. Other important confounders, including previous dengue infection, medication use (e.g., immunosuppressants or nephrotoxic agents), nutritional status, and access to timely healthcare, were not available in the dataset and may contribute to residual confounding. Third, CKD was identified from a clinician-reported comorbidity field in the dengue notification form, without access to baseline eGFR, albuminuria, or duration of kidney dysfunction, so some misclassification of CKD status is possible.

Nonetheless, the consistency of associations across multiple outcomes supports the conclusion that CKD is a strong and independent risk factor for adverse dengue-related events. The co-occurrence of comorbidities such as diabetes, hypertension, and hematologic disorder conditions known to be associated with immune dysregulation and endothelial dysfunction—may further amplify the vulnerability of CKD patients to poor outcomes during dengue infection. These comorbidities contribute to a pro-inflammatory state and vascular endothelial impairment, which can exacerbate the severity of infectious diseases, such as dengue. For instance, chronic diseases, including CKD, are recognized to share features of endothelial dysfunction, such as increased oxidative stress, systemic inflammation, and impaired endothelial repair mechanisms, all of which can compromise vascular integrity during infections [26].

## Practical implications

The findings of this study support the inclusion of CKD as a high-priority condition in dengue triage algorithms and national surveillance alerts. Given the significantly elevated risks of hospitalization, progression to severe dengue, and death, patients with CKD should be proactively identified during outbreaks and prioritized for early clinical evaluation. Additionally, clinicians caring for patients with CKD who develop dengue should recognize these individuals as clinically vulnerable and ensure timely assessment and follow-up, including careful monitoring of hemodynamic status and kidney function during the acute illness. Early and individualized fluid management may be particularly beneficial in minimizing renal deterioration while avoiding volume overload. Incorporating CKD risk stratification into outbreak response protocols may improve resource allocation, reduce morbidity, and ultimately prevent deaths among this vulnerable group. These findings strongly recommend that patients with CKD be classified as a high-risk group for prioritization in national dengue vaccination programs, especially in endemic areas like Brazil. In addition, tailored health education and dengue prevention strategies directed at people living with CKD, for example, within nephrology and dialysis services, may help ensure earlier care-seeking, adherence to warning signs, and uptake of vaccination in this high-risk group.

## Conclusion

In this nationwide cohort, pre-existing CKD was independently associated with higher odds of hospitalization, severe dengue, and death among patients with dengue fever in Brazil. These findings underscore the need to recognize individuals with CKD as a clinically vulnerable population during dengue outbreaks. Public health strategies should prioritize early identification, proactive monitoring, and tailored management of dengue in CKD patients to mitigate complications and reduce mortality. Furthermore, integrating nephrology care into infectious disease surveillance and outbreak preparedness frameworks may represent a critical step toward reducing the disproportionate burden of dengue in this high-risk group.

## Supporting information

S1 Table Operational definition of confirmed dengue in System for Notifiable Diseases (SINAN). (DOCX)
