# Supplementary material for: Adverse dengue outcomes in patients with chronic kidney disease: A population-based analysis of 5.8 million individuals from Brazil
Source: PLoS Negl Trop Dis. 2026 Jan 20;20(1):e0013927. doi: 10.1371/journal.pntd.0013927 (PMC12844498; doi:10.1371/journal.pntd.0013927)
Supplement: S1 Table — (DOCX) [file pntd.0013927.s001.docx]

**S1 Table. Operational definition of confirmed dengue in SINAN.** This table summarizes how “confirmed dengue” and “method of diagnosis” were operationalized in the SINAN dengue database, according to the Brazilian Ministry of Health dengue guideline (6th edition) and the official SINAN data dictionary.

| SINAN field / category | Operational definition in the analysis | Underlying guideline criteria (summary) |
| --- | --- | --- |
| Final classification = dengue (with or without warning signs, severe dengue) | Included as confirmed dengue if confirmation criterion = laboratory or clinical-epidemiological | Patient meets WHO/Ministry of Health clinical spectrum for dengue and is not discarded for alternative diagnosis. |
| Confirmation criterion = laboratory | Coded as “laboratory-confirmed” | At least one of: reactive NS1; positive viral isolation; detectable RT-PCR (≤5 days of symptoms); reactive IgM ELISA (≥6 days); or ≥4-fold rise in antibody titres in PRNT/IH with paired samples. |
| Confirmation criterion = clinical-epidemiological | Coded as “clinical-epidemiological confirmation” | Patient fulfils clinical case definition for dengue and has epidemiological link to a laboratory-confirmed case or resides in an area with documented dengue circulation, in the context of compatible symptoms and outbreak. |
| Confirmation criterion = discarded / under investigation / other | Excluded from the analytic cohort | Not considered confirmed dengue in SINAN; cases without final closure or with alternative diagnosis. |

The operationalization of “confirmed dengue” in this study follows the Brazilian Ministry of Health guideline *Dengue: diagnóstico e manejo clínico – adulto e criança* (6th ed.) and the SINAN dengue data dictionary. In practice, the field “final classification of case” defines whether the notification is dengue, while the field “criterion of confirmation/discard” specifies whether confirmation was based on laboratory or clinical-epidemiological criteria
